# Healthcare at Your Fingertips: The Acceptance and Adoption of Mobile Medical Treatment Services among Chinese Users

**DOI:** 10.3390/ijerph17186895

**Published:** 2020-09-21

**Authors:** Qingchuan Li

**Affiliations:** College of Humanity and Social Science, Harbin Institute of Technology, Shenzhen 518000, China; liqingchuan@hit.edu.cn

**Keywords:** mobile medical treatment services, user acceptance, personalization, interactivity, privacy concerns

## Abstract

Mobile health (mHealth) services have recently been receiving increasing attention. However, there is a lack of knowledge about how users accept and adopt mobile medical treatment (MMT) services, some of the most promising mHealth services that aim to extend the patient–physician relationship beyond the conventional clinic environment. To fill this research gap, this study proposes a research model for predicting consumers’ acceptance behavior toward MMT services based on the Technology Acceptance Model (TAM). A survey was conducted among 303 Chinese MMT service users to evaluate the proposed model and relevant hypotheses using partial least squares. Several key findings were summarized from the results: (1) the attitude toward using MMT, technology anxiety, and trust are significantly associated with users’ behavioral intention to use MMT services; (2) the perceived ease of use, perceived usefulness, and trust significantly influence users’ attitude toward using MMT services; (3) the perceived interactivity, perceived personalization, and privacy concerns have significant impacts on users’ perceptions of ease of use, usefulness, and trust toward MMT services. The current findings have both theoretical and practical implications that may guide practitioners and researchers to better understand consumers’ acceptance of MMT services.

## 1. Introduction

Patients typically visit physicians in person when they require professional medical advice. Such face-to-face communication facilitates the direct and personal connection between patients and physicians; however, this conventional context should also be considered in terms of its limitations [1]. For example, the awkward clinical setting, long waiting time, short consultation time, and high cost might limit the effectiveness of conventional face-to-face medical consultations [2,3]. Therefore, telecommunication systems have been implemented to deliver various medical treatment services at a distance, including urgent home care services, rural healthcare, prisoner health services, and psychiatry, which are widely known as telehealth or telemedicine [4]. With rapid technological advancements, electronic health (eHealth) later emerged as a more effective way to offer remote medical consultation and diagnosis by utilizing various kinds of information and communication technologies (ICTs) such as telecommunications and computers [5]. For instance, computer-based videoconferencing was proven to achieve the same effectiveness as face-to-face consultation when managing neurological symptoms [6] and psychiatry assessments [7]. Some other approaches involved email consultations or health portal systems to deliver follow-up treatments, access to lab test results, health data management, or treatment for nonurgent health issues [8]. Nevertheless, eHealth technologies present a number of shortcomings as well, especially for the medical services that require patient engagement. For example, eHealth systems generally require a certain level of computer expertise and considerable time and effort, with enrolment failure and usability problems being frequently reported [9,10].

With the ubiquity of mobile ICTs, mobile health (mHealth) applications—defined as “medical and healthcare solutions supported by mobile technologies such as mobile phones, smartphones, tablets, personal digital assistants and some other wireless technologies,” [4]—have recently gained increasing attention. Because of their high accessibility, portability, and low cost, mHealth applications can reach more users without location constraints and provide efficient healthcare responses in real time [11]. As reported, the size of the global mHealth market was US $46 billion in 2019 and it is projected to reach US $230 billion by 2027 [12]. In particular, China accounts for a substantial proportion of the mHealth market because of the increasing number of mobile Internet users in the country. Alongside the growth of the mobile ICT industry, the number of Chinese mobile Internet users reached 847 million in 2019 [13]. In the last decade, various services and platform providers have entered the mHealth market in China. Current mHealth applications offer plenty of functions, such as health data recording (e.g., physical examination report data, health evaluation data, and sports data), health-related information searching (e.g., information and reviews regarding hospitals, patients, and doctors), health-related knowledge bases, online shopping stores, health diagnosis and treatment, and hospital registration [14].

**Figure 1:**
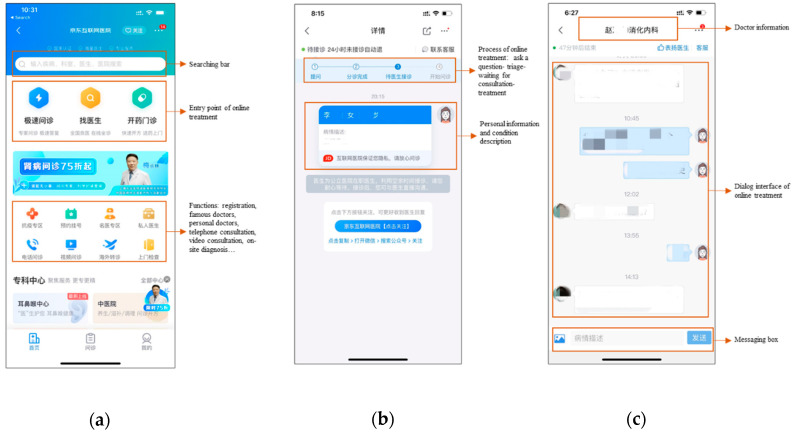
An example of an MMT application (Jingdong Health): (**a**) the home page; (**b**) the consultation waiting page; (**c**) the page for online treatment (https://app.jd.com/).

Particularly, the mobile medical treatment (MMT) service—a sub-function offered by mHealth applications—is a more promising remote medical treatment approach compared with the traditional face-to-face medical consultation, dial-up telecommunication treatment, and PC-based videoconferencing, because it could eliminate the time and space limitations of in-person doctor visits, improve patient engagement during the online medical treatment process, and further enhance the patient–physician interaction and communication opportunities outside the clinical setting [1,4]. In this study, the MMT service is defined as a sub-function that allows patients to seek professional and real-time medical treatment and diagnosis at any time and anywhere using mHealth apps installed on their smartphone or tablets and provides additional opportunities to extend patient–physician relationships beyond the conventional clinic environment. When using the MMT functions, patients can first choose the physician among thousands of registered health professionals and hospitals online according to the information provided. Patients will then access online treatment and diagnosis in the format of instant messaging. Next, they are asked to describe their diseases, symptoms, medical history, and other related information, while physicians are required to answer the questions within a given period. The MMT service offers various avenues of communication between patients and physicians, such as text-, picture-, and voice-based messages, as well as online voice and video chatting. In the Chinese market, typical mHealth apps that enable MMT services include Weixin Smart Hospital (https://weixin.qq.com/), Ali Health (https://www.alipay.com/), Ping An Good Doctor (https://www.jk.cn/), Good Doctor Online (https://www.haodf.com/), and Chunyu Doctor (https://www.chunyuyisheng.com/). Figure 1 shows an example interface of the MMT service provided by Jingdong Health (https://app.jd.com/).

Related research has extensively evaluated the acceptance and adoption of mHealth applications from a relatively broad perspective; these applications include various types of healthcare services focused on disease prevention, stress management, diabetes and weight loss, and health informatics and monitoring through smartphones, tablet computers, personal digital assistants, and wearable devices [11,15,16,17,18,19,20,21]. However, little is known about how users accept and adopt MMT services when using mHealth applications. Specifically, MMT services are believed to be quite different from other functions provided by mHealth applications because they enable instant communication between patients and physicians and thus have more flexibility in service delivery and increased interaction levels [22]. One study that partially explored users’ acceptance and adoption of online treatment services found that age, gender, and trust in general practitioners did not considerably influence users’ willingness to use an online treatment service [23]. Instead, it emphasized that users’ experience of online communication with physicians and their willingness to communicate with them online were key determinants of the adoption and purchase of online treatment services. Although this research uncovered interesting insights into online treatment services, it investigated a website-based treatment service rather than those provided by mHealth applications, which are the focus of the current study. There still exists a need to investigate the possible factors that inhibit patient–physician online communication and further hinder the acceptance and adoption of current MMT services. Accordingly, this study aimed to investigate Chinese users’ perception and usage behavior of MMT services and examine the possible facilitators and barriers that affect their acceptance of MMT services. To fulfill these purposes, this research attempts to answer the following research questions (RQs):

**RQ1**.  *How do Chinese users perceive and use the MMT services provided by current mHealth applications?*

**RQ2**.  *What factors may facilitate or hinder Chinese users’ acceptance and adoption of MMT services?*

**RQ3**.  *How does the patient–physician communication pattern influence Chinese users’ medical-care-seeking behavior?*

In the following sections, this article reviews the related literature and proposes the research model and hypotheses. It then elaborates on the methods including the instrument development, participant recruitment, and data collection and analysis. Then, the results are analyzed and discussed in detail, followed by the conclusions.

## 2. Literature Review and Hypothesis Development

The mHealth industry that provides MMT services is still in the phase of experimentation and exploration; thus, obtaining the public’s awareness and acceptance is crucial [24]. As mentioned above, numerous studies have investigated the factors that influence users’ acceptance and adoption of general mHealth functions, such as disease prevention and management [11,15,16,17,18,19,20,21], where the TAM is the most widely applied technology acceptance theory. The TAM regards behavioral intention as a sign of acceptance, which is influenced by users’ attitude toward relevant technologies. Specifically, this model focuses on the impacts of two factors: perceived usefulness and perceived ease of use. Perceived usefulness has a direct impact on an individual’s behavioral intention, and perceived ease of use directly influences perceived usefulness [25]. Overall, according to the results of related empirical and review studies, the TAM has been proven to be a robust and feasible model for studying users’ acceptance and adoption of health technologies [17,26].

An additional branch of study is dedicated to extending the TAM by exploring factors specific to health behavior, such as technology anxiety, privacy concerns, and perceived vulnerability and severity. For instance, technology anxiety and resistance to change are reportedly two key inhibitors affecting older people’s perceived ease of use and perceived usefulness of general mHealth services, factors that eventually influence their adoption intention [27]. Li et al. [28] combined the privacy calculus theory with the TAM to examine users’ acceptance and adoption of healthcare wearable devices. The results emphasized that an individual’s decision to adopt such devices depends on the trade-off between possible risks and benefits. In addition, a study found that factors derived from health behavior theories, namely perceived vulnerability and perceived severity, have significant impacts on users’ attitudes toward general mHealth services [26].

**Figure 2:**
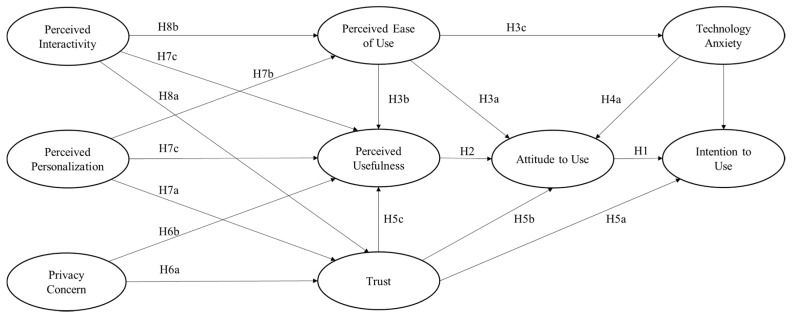
Research model of this study (Hypothesis denoted H).

Although the TAM has been proven to be supportive in predicting users’ acceptance and adoption of some general functions that provided by mHealth applications, few studies have evaluated its robustness in the context of MMT services. Considering that users tend to perceive a high level of uncertainty when experiencing the transition of medical consultation from offline to online, it is vital to identify the possible factors that may influence users’ acceptance and adoption of MMT services. Therefore, the current research proposes a research model that integrates the TAM constructs and a few external factors—including trust, privacy concerns, personalization, and interactivity—as shown in Figure 2.

### 2.1. TAM Constructs

Consistent with previous studies [25], the current research regards users’ intention to use services as a reflection of MMT applications’ acceptance and adoption, which is hypothesized to be influenced by users’ attitudes toward using the relevant services. In addition, users’ attitudes, which refers to their positive or negative feelings about using MMT services, are further influenced by two key constructs: perceived usefulness and perceived ease of use. Specifically, perceived usefulness indicates the degree to which individuals believe that using MMT services would improve their medical-care-seeking performance. Perceived ease of use, which refers to individuals’ perception that using MMT services is effortless, is also expected to have a direct impact on their perceived usefulness. Therefore, we proposed the following hypotheses:

**Hypothesis 1** **(H1).** *Individuals’ attitudes toward using MMT services positively affect their intention to use such services in the future.*

**Hypothesis 2** **(H2).** *Individuals’ perceived usefulness positively affects their attitudes toward using MMT services.*

**Hypothesis 3a** **(H3a).** *Individuals’ perceived ease of use positively affects their attitudes toward using MMT services.*

**Hypothesis 3b** **(H3b).** *Individuals’ perceived ease of use positively affects their perceived usefulness of using MMT services.*

The main factors in the TAM, namely perceived usefulness and perceived ease of use, are also determined by other antecedents. For instance, the United Theory of Acceptance and Use of Technology model extended the TAM with additional variables [29]. Particularly, technology anxiety, which pertains to the apprehension or discomfort which people experience when they think of using technology, is a major influential variable [30]. Empirical studies have identified a negative relationship between individuals’ technology anxiety and perceived ease of use [27,30,31]. Moreover, technology anxiety reportedly influences individuals’ overall levels of satisfaction and intention to use self-service technologies [32]. Within this research context, investigating users’ willingness to use online treatment services is also critical because such services represent a novel concept that may dramatically change the traditional way of seeing a doctor. Accordingly, we formulated the following hypotheses:

**Hypothesis 3c** **(H3c).** *Individuals’ perceived ease of use negatively affects their technology anxiety toward MMT services.*

**Hypothesis 4a** **(H4a).** *Technology anxiety negatively affects individuals’ attitudes toward using MMT services.*

**Hypothesis 4b** **(H4b).** *Technology anxiety negatively affects individuals’ intention to use MMT services.*

### 2.2. Trust, Privacy Concerns, Personality, and Interactivity

Users may hesitate to adopt an mHealth service because they are unfamiliar with the service provider and anxious regarding sharing their personal information. Therefore, the influence of trust on mHealth acceptance has attracted considerable attention in mHealth studies. Trust indicates a user’s perception of the trustworthiness of mHealth services [11,33] and has been well established as a major factor influencing users’ intentions of accepting and adopting mHealth services [11,34]. Trust also plays a vital role in reducing uncertainty when people use mHealth services: it induces a higher level of perceived usefulness and better attitude toward using mHealth services [19]. Thus, it is hypothesized that:

**Hypothesis 5a** **(H5a).** *Trust positively affects individuals’ intentions to use MMT services.*

**Hypothesis 5b** **(H5b).** *Trust positively affects individuals’ attitudes toward using MMT services.*

**Hypothesis 5c** **(H5c).** *Trust positively affects individuals’ perceived usefulness of MMT services.*

Privacy concerns, defined as the users’ authority to freely choose in which case and to what extent their personal information would be exposed to others [35], represent an additional factor that hampers users’ adoption of mHealth services through mobile platforms [36] and inhibits users’ trust in service providers [37,38]. When using MMT services, users may also feel apprehensive regarding the unauthorized access and unauthorized tracking of their personal information if the service provider could not fulfil their privacy protection responsibilities [37]. Hence, investigating the influence of privacy concerns on users’ perceived trust of MMT services is crucial. Privacy concerns have been reported to diminish users’ perceived benefits and usefulness of relevant services [39,40]. Accordingly, we propose the following hypotheses:

**Hypothesis 6a** **(H6a).** *Individuals’ privacy concerns negatively affect their perceived trust of MMT services.*

**Hypothesis 6b** **(H6b).** *Individuals’ privacy concerns negatively affect their perceived usefulness of MMT services.*

In addition to the aforementioned perceptual constructs, design features are also critical in predicting users’ acceptance of MMT services. The impact of personalization on users’ trust toward mobile commerce has been confirmed; a study reported that if customer services provided personalized and customized services according to users’ specific needs and characteristics, consumers would have a higher level of trust in the service provider and enhanced willingness to make purchases [41]. In addition, the impacts of personalization were also reported to be associated with users’ behavioral intentions of accepting general mHealth applications, in which the factor of trust played a mediating role [11]. Moreover, a service’s personalization or customization capabilities can also influence users’ perceived ease of use [42] and perceived usefulness [40,43] in the context of websites, high-tech products, and online customer services. Therefore, we hypothesize that:

**Hypothesis 7a** **(H7a).** *Individuals’ perceived personalization positively affects their perceived trust in MMT services.*

**Hypothesis 7b** **(H7b).** *Individuals’ perceived personalization positively affects their perceived ease of use of MMT services.*

**Hypothesis 7c** **(H7c).** *Individuals’ perceived personalization positively affects their perceived usefulness of MMT services.*

By providing more communication modes and instant feedback, mobile ICTs afford a higher level of interactivity between patients and physicians [22]; in this aspect, MMT services vary considerably from conventional eHealth services [4]. In mobile commerce, interactivity is defined as the extent to which users perceive that they have control over the interaction process with the system and how the system responds to the users’ communicative behavior [44]. Studies have revealed that the level of interactivity positively influences users’ trust [45], perceived ease of use, and perceived usefulness [46,47] when using mobile commerce services or government services. Although few studies have examined the effects of interactivity on mHealth service acceptance, a higher level of interactivity would likely be associated with improved patient–physician communication, which may further induce higher levels of trust, perceived ease of use, and perceived usefulness [44,45,46,47]. Hence, it is hypothesized that:

**Hypothesis 8a** **(H8a).** *The level of interactivity positively affects individuals’ perceived trust in MMT services.*

**Hypothesis 8b** **(H8b).** *The level of interactivity positively affects individuals’ perceived ease of use of MMT services.*

**Hypothesis 8c** **(H8c).** *The level of interactivity positively affects individuals’ perceived usefulness of MMT services.*

## 3. Methods

### 3.1. Instrument Development

To test the proposed hypotheses and conceptual model, we conducted a web-based survey comprising three sections: first, the participants’ socio-demographic information was collected (age, gender, education level, city of residence, and monthly income); second, the participants were asked about their previous usage experience with MMT services, including the mHealth app where they received the MMT services, the frequency with which they used MMT services, the registered departments, disease types, disease severity, and communication methods when using MMT services; third, the participants were asked to recall the last time that they used MMT services through their mHealth app and answer the questions formulated by the different instrument items represented in the research model, as shown in Table 1.

Specifically, the instrument items were derived from related studies. The measurements for intention to use, attitudes toward use, perceived usefulness, and perceived ease of use were derived from models related to the TAM [20,25,27,48,49]. The measurement for technology anxiety was adapted from [20,27,50,51], while the measurement for trust from [11,19,52,53]. The item measuring interactivity was derived from [46,54,55], the item measuring personalization was adapted from [11,41], and the measurement for privacy concerns was derived from [11,56]. To increase the research validity, two local experts with extensive experience in conducting user research supported the validation of these instrument items and offered some wording modifications. The survey was then pilot-tested with four MMT users of different ages and from different backgrounds and further revised in terms of written language and organization of questions according to their feedback. Table 1 lists the questionnaire items employed in this study. Each item was measured using a 5-point Likert scale ranging from “strongly disagree” (1) to “strongly agree” (5).

### 3.2. Participant Recruitment and Data Collection

The survey was documented and distributed through a Chinese online survey system, Wen Juan Xing (https://www.wjx.cn). Survey questions and options could be found in Supplementary Materials File S1. The whole study was approved by the office of Research Affairs of Harbin Institute of Technology, Shenzhen, and the respondents were informed that participation was not compulsory and that they were free to quit the survey at any time. The participants were required to be above 18 years old and have used MMT services provided by various mHealth apps at least once. In other words, all the participants should have consulted physicians about their diseases through MMT services in the past. Therefore, before the survey study, we reviewed the 16 highest-ranked mHealth apps that provide MMT services based on the top downloaded apps under the medical category from the iOS app store (https://www.apple.com/ios/app-store/), Huawei App Gallery (https://appgallery1.huawei.com/#/Featured), Mi store (http://m.app.mi.com/), as well as some news and market reports [58,59]. We summarized a few keywords—such as “ask the doctor”, “rapid inquiry”, “online diagnosis”, “professional inquiry”, and “emergency treatment”—that were frequently used to describe the MMT function in these mHealth apps. The participants were then asked to report whether they had used the MMT services that contain the aforementioned keywords.

The data collection was conducted in April 2020. In total, 303 valid questionnaires were received from 554 respondents (31 questionnaires were considered invalid because the respondents had no usage experience of MMT services and 220 questionnaires were disqualified based on the attention filter). A reward RMB 11 was paid to each respondent and the survey platform.

### 3.3. Data Analysis

A descriptive analysis was conducted on participants’ socio-demographic data (e.g., age, gender, education level, and monthly income), their usage behavior of MMT services (e.g., the mHealth app where participants received the MMT services, usage frequency of MMT services, registered departments, disease types, disease severity, and communication methods), and their evaluations in terms of perceived usefulness, perceived ease of use, technology anxiety, trust, perceived interactivity, perceived personalization, privacy concerns, and attitude toward and intention to use MMT services. Then, structural equation modeling was employed to test the hypotheses proposed in this research. Specifically, the research model was tested using partial least squares (PLS) for the following reasons: firstly, PLS can be used to assess the causal relationships among different stages and the layers of the model constructs by calculating the loadings and weights of construct indicators [60]; secondly, PLS is more appropriate for relatively small samples [61] and is more suitable for testing theoretical models that are developed at an early stage [60]. Thus, PLS was deemed ideal for the current study.

## 4. Results

### 4.1. Description of Respondents

The 303 respondents were from 27 provincial regions of China and 84.1% of them were citizens living in first-, second-, and third-tier cities, which may be because MMT services are still at the initial stage of development and city residents have greater access to this new form of medical consultation. The sample comprised 47.2% males and 52.8% females. Among the respondents, 24.1% were aged from 18 to 25 years, 57.1% from 26 to 35 years, 14.2% from 36 to 45 years, 3.9% from 46 to 55 years, and 0.7% were older than 56 years. Most of the respondents (95.1%) had at least an undergraduate degree, followed by those with a high school diploma (4.3%) and those who had completed middle school or below (0.6%). Additionally, 46.2% of the respondents had a monthly income between RMB 5000 and 10,000, followed by those earning less than RMB 5000 (26.7%), between RMB 10,000 and 15,000 (17.5%), and more than RMB 15,000 (9.6%). These statistics are basically consistent with the actual situation of the average education levels and monthly income of city residents living in first-, second-, and third-tier cities [62]. The respondents’ socio-demographic information is presented in Table 2.

The respondents’ usage behavior of the MMT services was investigated. Among the respondents, 66.0% reported having used the MMT services provided by the mHealth app of Ali Health to consult with doctors, followed by the apps of Ping An Good Doctor (60.1%), Wexin Smart Hospital (58.7%), Good Doctor Online (45.9%), Chunyu Doctor (34.0%), Jingdong Health (16.2%), Wedoctor (13.5%), Miaoshou Doctor (7.6%), Health 160 (6.9%), Daxiang Doctor (5.6%), and others (12.2%). Among them, 44.9%, 37.3%, and 14.2% of the respondents reported having used such services three to five times, more than five times, and one to two times, respectively; 3.6% were unable to recall their exact usage frequency. The respondents reported using the following online hospital departments: internal medicine (24.8%), surgery (19.1%), dermatology (16.5%), E.N.T (12.2%), stomatology (6.9%), pediatrics (6.6%), obstetrics and gynecology (5.6%), psychology (3.3%), ophthalmology (2.0%), orthopedics (0.7%), and others (2.3%). In particular, 59.6% of the respondents indicated that they were using MMT applications for minor infection, such as the flu, colds, or allergies; 24.4% of them utilized mHealth applications to consult with doctors regarding chronic diseases, such as diabetes and hypertension, and 16.0% of them used such applications to track their long-term health conditions after initial consultation and treatment using other methods. When communicating with physicians through MMT applications, 92.1% of the respondents employed text messaging, 67.3% used photo messaging, and 29.7% used voice messaging, followed by those who used voice chatting (20.8%), calling (12.9%), video chatting (9.2%), and group chatting (7.3%). In most cases, the respondents consulted with doctors regarding diseases of moderate severity (53.1%). Some of the participants consulted with doctors online regarding diseases that were not urgent (35.0%) or not urgent at all (2.0%), whereas 9.9% of them used online treatment applications for urgent disease enquiries.

### 4.2. Measurement Model

The composite reliability (CR) and average variance extracted (AVE) were employed to test the reliability of each construct and compare the consistency between the instrument measurements used in this research and those of previous studies. The CR values for the constructs formulated in this study ranged from 0.798 to 0.904 and were higher than the suggested cutoff value of 0.70 (Table 3) [63]. The AVE ranged from 0.553 to 0.703, also exceeding the suggested accepted value of 0.50 [63]. Thus, the results indicated well-constructed and reliable constructs and measurements (Table 3).

The convergent validity was measured according to the item loading, and any loading smaller than 0.70 was considered insufficient to measure the instrument construct [52,63]. The results indicated that all the construct items, except TRU4, had loadings greater than 0.70, ranging from 0.701 to 0.902 (Table 3). Thus, the results suggest that the instrument constructs have good convergent validity. Because the item loading for TRU4 was only slightly below 0.70, it was retained as a construct item.

The discriminant validity was examined to further consider the similarity of measurements between different construct pairs. As suggested by Fornell and Larcker [63], discriminant validity is assured when the square root of the AVE for a construct is larger than the correlations between the construct and other constructs in the research model. The square roots of all AVEs were greater than 0.740; this value is considerably higher than any of the correlations, whose maximum value is 0.711 (Table 4). Thus, the results satisfy the criteria of Fornell and Larcker [63] and suggest favorable discriminant validity.

### 4.3. Structural Model

**Figure 3:**
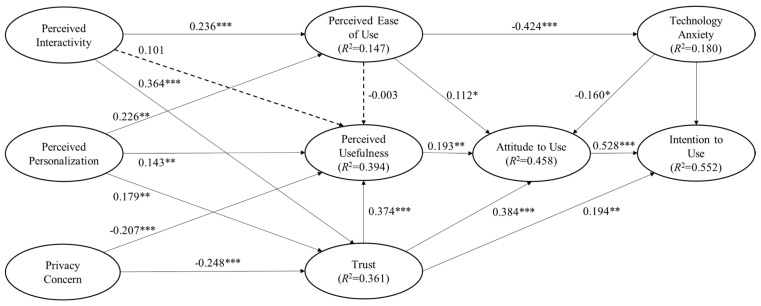
PLS results and significant path coefficients: * *p* < 0.05, ** *p* < 0.01, and *** *p* < 0.001.

A structural model was then developed to ascertain any explanatory relationships by using the PLS test. The PLS analysis results (Figure 3 and Table 5) revealed that the respondents’ attitudes toward use, trust, perceived ease of use, and technology anxiety accounted for 55.2% of the variance in their intention to use MMT services (*R*^2^ = 0.552); 45.8% of the variance in the respondents’ attitude toward using MMT services was explained by their perceived ease of use, perceived usefulness, trust, and technology anxiety (*R*^2^ = 0.458); 39.4% of the variance of the respondents’ perceived usefulness of MMT services could be explained by their perceived interactivity, perceived personalization, privacy concerns, trust, and perceived ease of use (*R*^2^ = 0.349); lastly, the respondents’ perceived interactivity, perceived personalization, and privacy concerns explained 36.1% of the variance in their trust in MMT services (*R*^2^ = 0.361). In addition, the variance in their perceived ease of use and technology anxiety was found to be explained by the variables in this research model (*R*^2^ = 0.147 and *R*^2^ = 0.179, respectively). Although these two values were slightly lower than the suggested coefficient of determination of 0.190 [64], they were regarded as acceptable considering the exploratory nature of the current research.

According to the PLS results, hypotheses H1 and H2 were supported; these hypotheses focused on the relationship between the participants’ attitude toward using and intention to use MMT services (*β* = 0.528, *t* = 8.876) and the relationship between their perceived usefulness and attitude toward using MMT services (*β* = 0.193, *t* = 3.543). The results also suggest that the participants’ perceived ease of use significantly influenced their attitude toward using MMT services (*β* = 0.112, *t* = 2.031) and their corresponding technology anxiety (*β* = −0.424, t = 9.400), supporting H3a and H3c. However, H3b was not supported by the results. H4a and H4b were supported because technology anxiety was found to significantly influence the participants’ attitude toward using (*β* = −0.160, *t* = 2.360) and intention to use (*β* = −0.128, *t* = 2.308) MMT services. Trust had a significant impact on the respondents’ intention to use (*β* = 0.194, *t* = 3.452), attitude toward using (*β* = 0.384, *t* = 5.623), and perceived usefulness of (*β* = 0.374, *t* = 5.863) MMT services, supporting H5a–H5c.

Respondents’ privacy concerns significantly affected their trust in (*β* = −0.248, *t* = 4.750) and perceived usefulness of (*β* = −0.207, *t* = 3.975) MMT services (supporting H6a and H6b). Perceived personalization was found to significantly influence the respondents’ trust in (*β* = 0.179, *t* = 3.329), perceived ease of use of (*β* = 0.226, *t* = 3.375), and perceived usefulness of (*β* = 0.143, *t* = 2.650) MMT services (supporting H7a–H7c). Lastly, perceived interactivity had significant effects on the respondents’ trust (*β* = 0.364, *t* = 6.280) and perceived ease of use (*β* = 0.236, *t* = 4.141) (supporting H8a and H8b); however, H8c was not supported by the results.

## 5. Discussion

MMT services are popular in the field of mHealth. With the aim of extending the patient–physician relationship beyond the conventional clinic setting, MMT raises many questions concerning users’ adoption of such services, the factors that may hinder their acceptance of these services, and how the novel communication pattern might change users’ medical consultation behavior. To address these questions, we constructed an exploratory model to study Chinese users’ MMT service acceptance behavior. Firstly, the results provided insights into users’ current usage and adoption behavior of MMT services. Secondly, the results emphasized the facilitators and inhibitors of users’ adoption behavior toward MMT services, with a specific focus on privacy concerns, personalization, and interactivity. Furthermore, although the current findings should be viewed in terms of their theoretical and practical contributions, their possible limitations should also be considered.

### 5.1. Insights into Users’ Adoption Behavior toward MMT Services

The Chinese mobile industry has experienced a great expansion in mHealth services and users have become accustomed to obtaining medical advice and consultations from the MMT services provided by mHealth applications. Ali Health, Ping An Good Doctor, Winxin Smart Hospital, Good Doctor Online, and Chunyu Doctor are the most frequently used mHealth apps where users could get access to MMT services in the Chinese market according to this research, with a usage rate between 34.0% and 66.0%. Most of the respondents (82.2%) in this study had used MMT services more than three times to address minor infections (50.6%) or chronic diseases (24.4%). Some users also utilized MMT services to track their health conditions after in-person clinic visits. Users tended to resort to MMT services to obtain treatment for diseases that were not urgent (35.0%) or of moderate severity (53.1%). Furthermore, relative to conventional approaches, such as online doctor review [65,66] and Question and Answer platforms [1], MMT services provide multiple communicative modalities—text messaging, picture messaging, voice messaging, voice chatting, making calls, video chatting, and group chatting—which enhance the patients’ feelings of “connected presence” [22]. Thus, MMT services could deliver more real-time and intimate medical treatment and diagnosis services outside the clinic environment due to their instant messaging features, which could compensate for the lack of face-to-face communication in the online environment and further improve patient–physician communication effectiveness [1,52].

### 5.2. Factors Influencing the Acceptance of MMT Applications

The TAM is a validated and robust research model for predicting users’ acceptance and adoption of health information technologies [17]. Similar to previous studies that applied the TAM in the context of general mHealth applications [21,49,67,68], the current results emphasized that the users’ perceived usefulness and perceived ease of use together significantly influenced their attitude toward using MMT services, which further affected their intention to use such services in the future. However, in contrast to the findings of some previous studies [21,68,69,70], the present work found that users’ perceived ease of use of MMT services did not have a significant influence on their perceived usefulness. This might be because users who have already experimented with MMT services do not regard ease of use as a major consideration when weighing possible benefits and risks brought by such services. Venkatesh et al. [29] also reported that perceived ease of use only plays a vital role in users’ pre-implementation phase and not so much in the post-implementation phase during technology acceptance.

The current results also highlight the necessity of integrating additional variables, such as technology anxiety and trust, into the TAM to maximize its predicting power. Technology anxiety, which concerns users’ fear and apprehension when using technologies, was reported to be particularly critical for older users’ acceptance and adoption of mHealth applications, which is because older adults are more hesitant to change their medical-care-seeking behavior and lifestyles [18]. In the same vein, although this research did not study users’ acceptance and adoption behavior toward MMT services among older adults, it found that technology anxiety can negatively affect users’ intentions to use and the perceived usefulness of MMT services [32]. This may be because the newly introduced MMT service proposes a dramatic change from the conventional style of face-to-face clinic visits to online-only medical treatment services. In addition, the current results also agree with those of previous studies claiming that users are less likely to harbor negative feelings when they perceive technologies as easier to use [27,30,31].

Trust is another variable that is frequently integrated into the TAM in various technological domains, such as e-commerce, online shopping, and eHealth. The current study confirmed that trust has significant positive impacts on improving users’ perceived usefulness and attitude toward using MMT services. These results are consistent with those of previous studies that revealed that trust can help to reduce uncertainty when users adopt new types of services [19,36] and further determines users’ intention to use MMT services in the future [11,19,34]. By persuading themselves that the technology could meet their expectations, the resulting increased trust is believed to reduce users’ perceived complexity and uncertainty when dealing with this lifestyle transition [17].

To further identify the enablers and inhibitors of users’ affective outcomes, possible influential factors related to users’ privacy concerns, perceived personalization, and perceived interactivity were examined. Firstly, although mobile ICTs enable a higher level of flexibility and convenience, they are more likely to trigger consumers’ privacy concerns. This research found that users’ privacy concerns had significant negative influences on their perceived usefulness and trust in MMT services. This finding agrees with that of previous studies that emphasized the impact of trust on users’ acceptance of various technology settings, such as commerce websites [40], general mHealth services [11], and wearable devices [28]. In particular, when consumers seek personalized and interactive medical treatment services online, information privacy is a major factor that increases their perceived risk of disclosing personal information and reduces the possible benefits to be gained from relevant services.

In the current study, the level of perceived personalization was found to significantly improve users’ perceived ease of use, perceived usefulness, and trust in terms of MMT application acceptance and adoption. These results are consistent with those of related studies that emphasized the effects of personalization in the context of mobile commerce [41] and general mHealth services [1]. The ability of mobile ICTs to more effectively collect personal health information (e.g., locations, preferences, living habits, and health status) enables service providers to offer more personalized healthcare services. Thus, users are more likely to regard MMT applications as easy to use, helpful, and trustworthy.

Interactivity is essential to users’ ICT service acceptance, particularly in e-commerce [45,46,71], e-government [72,73], and e-learning [55]. However, little research has investigated the role of interactivity in mHealth’ acceptance, except for one study that reported that the user experience and willingness to communicate with the physicians may positively influence their willingness to use the web-based treatment services [23] and another study that proposed that healthcare professionals’ perceived interactivity of health information technologies may negatively influence the perceived threat to their professional autonomy [69]. The results of the current study addressed this research gap by identifying that users’ perceived interactivity significantly and positively influences their perceived ease of use and trust in MMT services, which is consistent with the findings of a related study conducted through a general website [47]. This may be because MMT services offer instant messaging between patients and physicians, which provides consumers with the perceptual illusion of being with their physician to the point that it resembles face-to-face clinic visits [22,71]. This results in users having a higher level of perceived ease of use and trust in MMT services. Nevertheless, perceived interactivity was found to have no significant influence on users’ perceived usefulness, indicating that interactivity did not necessarily contribute to users’ perceived benefits and usefulness of MMT applications.

### 5.3. Implications

The results of the current study have both theoretical and practical value. Theoretically, this study validated the robustness of the TAM in predicting users’ acceptance and adoption intention of MMT services. Compared with previous studies that focused on general eHealth and mHealth services [17,70], the current findings more precisely reflect the acceptance behavior for online medical treatment in the field of mHealth. Furthermore, the findings identified the significant influences of additional factors, such as technology anxiety, trust, privacy concerns, personalization, and interactivity, which highlight the necessity of extending the TAM by emphasizing these unique variables and features in the context of mHealth. In this manner, we proposed an extended TAM model that could better explain the variance in users’ acceptance and adoption behavior in the specific mHealth subdivision of MMT services.

Practically, by identifying the impacts of privacy concerns, personalization, and interactivity on users’ trust, perceived ease of use, and perceived usefulness, the findings provide valuable insights for mHealth application designers and practitioners. Firstly, MMT service providers should pay attention to both service personalization and information sensitivity [28]. Given the positive impact of personalization on users’ perceived ease of use, perceived usefulness, and trust, mHealth applications should offer personalized MMT services according to users’ interests and preferences. For example, MMT services could provide search tools, category options, filtered content, and doctor and hospital overviews according to the users’ location, medical history, or budget; this would enable the applications to meet the customers’ needs and improve the efficiency of online medical treatment services.

Previous studies have reported that privacy–personalization paradox factors can affect user acceptance behavior; users may be concerned about privacy leaks because service personalization inherently requires the disclosure of personal information [11,40,74,75]. Therefore, a high level of personalization does not necessarily ensure mHealth acceptance. Considering the negative impacts of privacy concerns on users’ perceived usefulness and trust reported by the current study, MMT providers are advised to provide detailed explanations of how users’ personal information is utilized and guarantee that all the obtained personal information is secure and that the provider rejects function creep. Moreover, MMT service providers should take special care with those who may have more privacy concerns and offer them alternative ways to communicate their personal needs. For instance, MMT applications could allow users to provide only basic information and revoke their personal information at any time during the service [76].

To our knowledge, this study is one of the first attempts to specifically investigate the possible impacts of interactivity on MMT services. The current findings suggest that users’ perceived interactivity influences their perceived ease of use and trust in relevant services. Hence, it emphasizes the importance of enhancing the user interactivity and engagement when using MMT services. Possible strategies include providing more diverse channels of information exchange between patients and physicians, enabling medical and health-related data tracking, such as graphic or gamified displays of health records, and employing virtual or conversational assistants to enable more efficient feedback [77]. Future research should evaluate the effectiveness of the aforementioned strategies when applied in real-world MMT services.

### 5.4. Limitations and Future Work

Despite the previously mentioned implications, this study should be considered in terms of its potential limitations. Firstly, although the survey data used in the current study were gathered from a survey distribution website and the respondents, who received compensation, covered a wide range of locations and age groups, the time period for data collection was relatively short, which may influence the external validity of the findings. Future research could distribute the surveys over a longer period and gather data from more distribution platforms. Secondly, this study considered behavioral intention rather than actual usage behavior as a proxy for acceptance of MMT services. Follow-up studies should investigate users’ actual acceptance behavior toward MMT services in terms of how users continue to use such applications over time. Thirdly, the respondents in this study comprised Chinese users. Most of them were young city residents from first-, second-, and third-tier cities of China, with a high education level and monthly income. They habitually used smartphones or other mobile ICTs for social communication, e-commerce, and other life service applications. Therefore, the results could not be generalized to all user groups. Future studies should further evaluate the proposed research model by considering differences in age, education level, income, cultural background, and technological expertise. Furthermore, the Chinese mHealth apps that provide MMT services are still in the early stage of exploration. Most of them did not provide systems of electronical medical records (EMR) for physicians to use. Consequently, the physicians do not have access to patients’ previous medical, medication, or family history records, and they are not required to keep the treatment and medical records in the backend systems of mHealth apps. They usually ask the patients about their symptoms and medical history by routine inquiry or the patients’ self-reports at the first time of consultation or review the text-, voice-, or picture-based chatting history in relevant mHealth apps for follow-up treatments. Thus, MMT services mostly represent as an approach to initial diagnosis and treatment of minor illnesses or long-term health condition monitoring of chronic diseases at the current stage. Extensive efforts are still needed in order to improve the MMT service quality in terms of the EMR’s development and management, introduction of medical insurance, the systems’ confidentiality and privacy issues, and so forth. The results should be further evaluated in the countries and areas with different ethical standards for healthcare services. In addition, this study mainly focused on the user acceptance and adoption of MMT services through mHealth apps installed on smartphones or tablets. Further studies are still needed to investigate how users accept and adopt such services when using wearable devices or other kinds of ICTs.

## 6. Conclusions

Despite the fact that mHealth applications have recently attracted increased attention, few studies have focused on how users accept and adopt MMT services. The current study is one of the first attempts to address research questions concerning how consumers accept and adopt MMT services and what factors facilitate and hinder their acceptance behavior toward MMT services. A research model was proposed to address the aforementioned research questions. The results indicate that the TAM remains a robust theoretical model for predicting consumers’ acceptance of MMT services. The current findings also emphasize the impact of some additional factors on users’ acceptance behavior toward MMT applications, including technology anxiety and trust. Furthermore, it is demonstrated that the introduction of MMT services may inevitably change the patient–physician relationship. Specifically, the instant messaging features provided by MMT services enable multiple communicative modalities between patients and physicians. Thus, the design features related to interactivity were found to significantly influence users’ perceived ease of use and trust of MMT services. At the same time, the transition from face-to-face clinic visits to online treatment services may induce anxiety in consumers regarding their personal privacy, which emphasizes the significant impact of users’ privacy concerns on their perceived usefulness and trust in MMT services. Overall, the findings of this research have both theoretical and practical implications. Further studies should be conducted to evaluate the effects of each antecedent that influences users’ perceptual and affective attributes regarding MMT applications in various usage contexts.

## Figures and Tables

**Table 1:** Construct items of the instrument.

Instrument Items		Questions	References
Intention to use	ITU1	I intend to use MMT services in the future.	[20,25,57]
	ITU2	I believe I will use MMT services in the future.	
	ITU3	I plan to use MMT services in the future.	
Attitude toward use	ATT1	Using MMT services is a good idea.	[25,48]
	ATT2	Using MMT services is a wise idea.	
	ATT3	I like using MMT services.	
Perceived usefulness	PU1	MMT services are suitable for solving my health problems.	[25,27,49]
	PU2	MMT services are effective for solving my health problems.	
	PU3	When using MMT services, my health problems are more likely to be resolved.	
Technology anxiety	TA1	I feel apprehensive about using MMT services.	[20,27,50,51]
	TA2	It scares me to think that I could cause the mobile device to induce bad consequences due to wrong operation.	
	TA3	I hesitate to use technology for fear of making mistakes I cannot correct.	
	TA4	I find MMT services somewhat intimidating.	
Perceived ease of use	PEOU1	Learning to operate MMT services will be easy for me.	[27]
	PEOU2	I can easily become skillful at using MMT services.	
	PEOU3	I can use MMT applications effectively to achieve my specific goals.	
	PEOU4	Overall, MMT services are easy to use.	
Trust	TRU1	This MMT service provider is trustworthy.	[11,19,52,53]
	TRU2	This MMT service provider provides reliable information.	
	TRU3	This MMT service provider keeps promises and commitments.	
	TRU4	This MMT service provider’s behavior meets my expectations	
Interactivity	INT1	Interacting with this MMT system is similar to having a conversation with a sociable, knowledgeable and warm representative from the company.	[46,54,55]
	INT2	I felt that this MMT system talked back to me while I was navigating.	
	INT3	I perceive the MMT system to be sensitive to my information requirements.	
	INT4	My interaction level with the MMT system was high.	
	INT5	I did not interact much with the system much.	
Personalization	PS1	By disclosing my information, the MMT service provider can understand my needs.	[11,41]
	PS2	By disclosing my information, the MMT service provider can know what I require.	
	PS3	By disclosing my information, the mHealth service provider will take my needs as its own preferences.	
Privacy concerns	PC1	My use of MMT services would make me lose control over the privacy of my information.	[11,56]
	PC2	Using MMT services would not cause any privacy problems.	
	PC3	Signing up for and using MMT services would lead to a loss of privacy for me because my personal information could be used without my knowledge.	
	PC4	Others might take control of my information if I use MMT services.	

**Table 2:** Respondents’ demographic information (*N* = 303).

		Frequency	Percentage (%)
Gender	Male	143	47.2
	Female	160	52.8
Age	18–25	73	24.1
	26–35	173	57.1
	36–45	43	14.2
	46–55	12	3.9
	Above 56	2	0.7
Education level	Primary school	1	0.3
	Middle school	1	0.3
	High school	13	4.3
	Undergraduate	252	83.2
	Postgraduate and above	36	11.9
Monthly income (RMB)	Below 5000	81	26.7
	5000–10,000	140	46.2
	10,000–15,000	53	17.5
	Above 15,000	29	9.6

**Table 3:** Means, reliability, and convergent validity.

Construct	Items	Mean (SD)	Standardized Factor Loading	CR	AVE
Intention to use	ITU1	4.3 (0.65)	0.871	0.860	0.673
	ITU2	4.3 (0.71)	0.812		
	ITU3	4.3 (0.70)	0.776		
Attitude toward use	ATT1	4.3 (0.56)	0.721	0.798	0.569
	ATT2	4.3 (0.69)	0.721		
	ATT3	4.1 (0.77)	0.816		
Perceived usefulness	PU1	3.9 (0.74)	0.798	0.801	0.573
	PU2	3.8 (0.84)	0.769		
	PU3	3.9 (0.73)	0.701		
Technology anxiety	TA1	2.1 (0.81)	0.825	0.853	0.593
	TA2	2.2 (1.00)	0.769		
	TA3	2.1 (0.90)	0.766		
	TA4	1.7 (0.66)	0.715		
Perceived ease of use	PEOU1	4.4 (0.70)	0.797	0.855	0.596
	PEOU2	4.4 (0.73)	0.786		
	PEOU3	4.1 (0.73)	0.785		
	PEOU4	4.3 (0.70)	0.716		
Trust	TRU1	4.1 (0.67)	0.798	0.831	0.553
	TRU2	4.1 (0.77)	0.753		
	TRU3	4.1 (0.72)	0.736		
	TRU4	3.9 (0.72)	0.684		
Perceived interactivity	INT1	3.8 (0.78)	0.775	0.842	0.572
	INT2	4.0 (0.80)	0.774		
	INT3	3.8 (0.79)	0.752		
	INT4	2.2 (0.82)	0.723		
Perceived personalization	PS1	4.0 (0.58)	0.839	0.822	0.698
	PS2	4.0 (0.77)	0.832		
Privacy concerns	PC1	2.7 (0.91)	0.902	0.904	0.703
	PC2	3.0 (0.96)	0.843		
	PC3	3.0 (0.98)	0.817		
	PC4	3.2 (1.00)	0.789		

Note: CR represents composite reliability and AVE represents average variance extracted.

**Table 4:** Discriminant validity.

	ITU	ATT	PU	TA	PEOU	TRU	INT	PS	PC
ITU	***0.820***								
ATT	0.711	***0.754***							
PU	0.482	0.511	***0.757***						
TA	−0.485	−0.488	−0.439	***0.770***					
PEOU	0.323	0.386	0.269	−0.424	***0.772***				
TRU	0.587	0.620	0.565	−0.511	0.401	***0.744***			
INT	0.527	0.569	0.427	−0.368	0.322	0.524	***0.756***		
PS	0.327	0.352	0.358	−0.304	0.315	0.366	0.376	***0.836***	
PC	−0.369	−0.455	−0.430	0.474	−0.218	−0.421	−0.375	−0.202	***0.839***

Note: The bold data on the diagonal are the square roots of AVE.

**Table 5:** Results of hypothesis testing.

Hypothesis	Path	Path Coefficient	*t*-Value	Supported
H1	ATT → ITU	0.528	8.876 ***	Yes
H2	PU → ATT	0.193	3.543 **	Yes
H3a	PEOU → ATT	0.112	2.031 *	Yes
H3b	PEOU → PU	−0.003	0.064	No
H3c	PEOU → TA	−0.424	9.400 ***	Yes
H4a	TA → ATT	−0.160	2.360 *	Yes
H4b	TA → ITU	−0.128	2.308 *	Yes
H5a	TRU → ITU	0.194	3.452 **	Yes
H5b	TRU → ATT	0.384	5.623 ***	Yes
H5c	TRU → PU	0.374	5.863 ***	Yes
H6a	PC → TRU	−0.248	4.750 ***	Yes
H6b	PC → PU	−0.207	3.975 ***	Yes
H7a	PS → TRU	0.179	3.329 **	Yes
H7b	PS → PEOU	0.226	3.375 **	Yes
H7c	PS → PU	0.143	2.650 **	Yes
H8a	INT → TRU	0.364	6.280 ***	Yes
H8b	INT → PEOU	0.236	4.141 ***	Yes
H8c	INT → PU	0.101	1.580	No

Note: * *p* < 0.05; ** *p* < 0.01; *** *p* < 0.001.

## Supplementary Materials

The following are available online at https://www.mdpi.com/1660-4601/17/18/6895/s1, Figure S1: Survey on Acceptance and Adoption Behavior of Mobile Medical Treatment Services.
