# Supplementary material for: Healthcare at Your Fingertips: The Acceptance and Adoption of Mobile Medical Treatment Services among Chinese Users
Source: Int J Environ Res Public Health. 2020 Sep 21;17(18):6895. doi: 10.3390/ijerph17186895 (PMC7558837; doi:10.3390/ijerph17186895)
Supplement: Supplementary file 1 [file ijerph-17-06895-s001.pdf]

File S1: Survey on Acceptance and Adoption Behavior of Mobile Medical Treatment Services

You are invited to participate in a study conducted by Qingchuan Li, an Assistant Professor of the School of Humanity and Social Science in the Harbin Institute of Technology, Shenzhen. The project has been approved by the office of Research Affairs of Harbin Institute of Technology, Shenzhen.

The aim of this study is to investigate the Chinese users' acceptance and adoption of mobile medical treatment services and identify possible factors that influencing users' acceptance and adoption behavior. If you are aged over 18-year-old and have previous experience of using mobile medical treatment services, you are eligible to participate. In this study, you will be asked to complete an online survey, which will take you about 5 to 20 minutes.

The survey should not result in any undue discomfort. The survey is completely anonymous, and all information related to you will remain confidential and will be identifiable by codes only known to the researcher. You have every right to withdraw from the study before or during the measurement without penalty of any kind. If you would like to obtain more information about this study, please contact Qingchuan Li by email, liqingchuan@hit.edu.cn.

Thank you for your interest in participating in this study.

Part I: Usage Experience

Have you ever used the mobile medical treatment services (hereinafter referred to as MMT services) provided by mHealth apps for online consultation?

For example, by sending text-, pictures-, voice-based messages, or through voice and video chat, you have consulted with online doctors through mHealth apps. The keywords of such services and functions usually include "ask the doctor", "rapid inquiry", "online diagnosis", "professional inquiry", and "emergency treatment".

☐Yes ☐ No

Part II Demographic Information

1. Gender： ☐ Male ☐ Female
2. Age： ________
3. Level of education：
   ☐ Primary school ☐ Middle school ☐ High school ☐ Undergraduate ☐ Postgraduate and above
4. The city where you currently live： ________
5. Monthly income (RMB)：
   ☐ Below 2000 ☐ 2000 – 5000 ☐ 5000 – 10000 ☐ 10000 – 15000 ☐ 15000 and above

Part III Usage Behavior

The following questions ask about your usage behavior with the MMT services. Please choose the option that best suits your personal situation:

1. Please choose the mHealth app where you received the MMT services：

   ☐ Weixin Smart Hospital

   ☐ Ali Health

   ☐ Jingdong Health

   ☐ Ping An Good Doctor

   ☐ Chunyu Doctor

   ☐ Good Doctor online

   ☐ Wedoctor

   ☐ Health160

   ☐ Miaoshou Doctor

   ☐ Yihe Health

   ☐ Daxiang Doctor

   ☐ Zhuozheng Health

   Others: ________

2. How many times have you used MMT services：

   ☐ 1-2 times ☐ 2-5 times ☐ More than 5 times ☐ I'm not sure

   Please recall the last time you used the above mHealth apps for online consultation and answer the following questions:

3. Your registered department：

   ☐ Internal medicine ☐ Surgery ☐ E.N.T ☐ Dermatology ☐ Obstetrics and gynecology ☐ Psychology ☐ Ophthalmology ☐ Orthopedics ☐ Pediatrics ☐ Stomatology ☐ Others:________

4. Your disease type：

   ☐ Minor infection, such as the flu, colds, or allergies ☐ Chronic diseases, such as diabetes and hypertension

   ☐ Tracking long-term health conditions after initial consultation and treatment using other methods

   ☐ Others: _______

5. The severity of your disease：

   ☐ Not urgent at all ☐ Not urgent ☐ Moderate ☐ Urgent ☐ Very urgent

6. When you are using the above mHealth apps to consult with doctors online, which of the following communication methods did you choose?

   ☐ Text messaging ☐ Photo messaging ☐ voice messaging ☐ Calling ☐ Voice chatting ☐ Video chatting ☐ Group chatting Others:________

Part IV Acceptance and Adoption Behavior

The following questions ask about your user experience and attitude with the MMT services. There is no right or wrong answer, please choose the option that best suits your actual situation:

**Perceived Usefulness of MMT services**					
1. MMT services are suitable for solving my health	Strongly disagree	Disagree	Neutral	Agree	Strongly agree

Item	Strongly disagree	Disagree	Neutral	Agree	Strongly agree
problems.	☐	☐	☐	☐	☐
2. MMT services are effective for solving my health problems.	☐	☐	☐	☐	☐
3. When using MMT services, my health problems are more likely to be resolved.	☐	☐	☐	☐	☐
**Perceived Ease of Use of MMT services**					
4. Learning to operate MMT services will be easy for me.	☐	☐	☐	☐	☐
5. I can easily become skillful at using MMT services.	☐	☐	☐	☐	☐
6. I can use MMT applications effectively to achieve my specific goals.	☐	☐	☐	☐	☐
7. Overall, MMT services are easy to use.	☐	☐	☐	☐	☐
**Trust with MMT services**					
8. This MMT service provider is trustworthy.	☐	☐	☐	☐	☐
9. This MMT service provider provides reliable information.	☐	☐	☐	☐	☐
10. This MMT service provider keeps promises and commitments.	☐	☐	☐	☐	☐
11. This MMT service provider's behavior meets my expectations.	☐	☐	☐	☐	☐
**Technology anxiety of MMT services**					
12. I feel apprehensive about using MMT services.	☐	☐	☐	☐	☐
13. It scares me to think that I could cause the mobile device to induce bad consequences due to wrong operation.	☐	☐	☐	☐	☐

14. I hesitate to use technology for fear of making mistakes I cannot correct.	Strongly disagree ☐	Disagree ☐	Neutral ☐	Agree ☐	Strongly agree ☐
15. I find MMT services somewhat intimidating.	Strongly disagree ☐	Disagree ☐	Neutral ☐	Agree ☐	Strongly agree ☐
**Privacy concerns of MMT services**					
16. My use of MMT services would make me lose control over the privacy of my information.	Strongly disagree ☐	Disagree ☐	Neutral ☐	Agree ☐	Strongly agree ☐
17. Using MMT services would not cause any privacy problems.	Strongly disagree ☐	Disagree ☐	Neutral ☐	Agree ☐	Strongly agree ☐
18. Signing up for and using MMT services would lead to a loss of privacy for me because my personal information could be used without my knowledge.	Strongly disagree ☐	Disagree ☐	Neutral ☐	Agree ☐	Strongly agree ☐
19. Others might take control of my information if I use MMT services.	Strongly disagree ☐	Disagree ☐	Neutral ☐	Agree ☐	Strongly agree ☐
**Personalization of MMT services**					
20. By disclosing my information, the MMT service provider can understand my needs.	Strongly disagree ☐	Disagree ☐	Neutral ☐	Agree ☐	Strongly agree ☐
21. By disclosing my information, the MMT service provider can know what I require.	Strongly disagree ☐	Disagree ☐	Neutral ☐	Agree ☐	Strongly agree ☐
22. By disclosing my information, the mHealth service provider will take my needs as its own preferences.	Strongly disagree ☐	Disagree ☐	Neutral ☐	Agree ☐	Strongly agree ☐
**Perceived interactivity of MMT services**					
23. Interacting with this MMT system is similar to having a conversation with a sociable, knowledgeable and warm representative from the company.	Strongly disagree ☐	Disagree ☐	Neutral ☐	Agree ☐	Strongly agree ☐
24. I felt that this MMT system talked back to me while I was navigating.	Strongly disagree ☐	Disagree ☐	Neutral ☐	Agree ☐	Strongly agree ☐
25. I perceive the MMT system to be	Strongly disagree	Disagree	Neutral	Agree	Strongly agree

	sensitive to my information requirements.	☐	☐	☐	☐	☐
26.	My interaction level with the MMT system was high.	Strongly disagree ☐	Disagree ☐	Neutral ☐	Agree ☐	Strongly agree ☐
27.	I did not interact much with the system much.	Strongly disagree ☐	Disagree ☐	Neutral ☐	Agree ☐	Strongly agree ☐
**Attitude toward using MMT services**						
28.	Using MMT services is a good idea.	Strongly disagree ☐	Disagree ☐	Neutral ☐	Agree ☐	Strongly agree ☐
29.	Using MMT services is a wise idea.	Strongly disagree ☐	Disagree ☐	Neutral ☐	Agree ☐	Strongly agree ☐
30.	I like using MMT services.	Strongly disagree ☐	Disagree ☐	Neutral ☐	Agree ☐	Strongly agree ☐
**Intention to use MMT services**						
31.	I intend to use MMT services in the future.	Strongly disagree ☐	Disagree ☐	Neutral ☐	Agree ☐	Strongly agree ☐
32.	I believe I will use MMT services in the future.	Strongly disagree ☐	Disagree ☐	Neutral ☐	Agree ☐	Strongly agree ☐
33.	I plan to use MMT services in the future.	Strongly disagree ☐	Disagree ☐	Neutral ☐	Agree ☐	Strongly agree ☐

The survey is completed. Thank you for your participation!
